# The role of TLR2 in the host response to pneumococcal pneumonia in absence of the spleen

**DOI:** 10.1186/1471-2334-12-139

**Published:** 2012-06-21

**Authors:** Adriana J J Lammers, AlexanderPNA de Porto, Onno J de Boer, Sandrine Florquin, Tom van der Poll

**Affiliations:** 1 Center of Infection and Immunity Amsterdam (CINIMA) Academic Medical Center, University of Amsterdam, Meibergdreef 9, 1105 AZ, Amsterdam, The Netherlands; 2 Center for Experimental and Molecular Medicine Academic Medical Center, University of Amsterdam, Meibergdreef 9, 1105 AZ, Amsterdam, The Netherlands; 3 Department of Pathology Academic Medical Center, University of Amsterdam, Meibergdreef 9, 1105 AZ, Amsterdam, The Netherlands; 4 Room G2-105, CEMM, Academic Medical Center, Meibergdreef 9, 1105 AZ, Amsterdam, The Netherlands

**Keywords:** *Streptococcus pneumonia*, Toll-like receptor 2, Splenectomy, Pneumonia

## Abstract

**Background:**

Asplenic individuals are susceptible for overwhelming infection with *Streptococcus pneumoniae*, carrying a high mortality. Although Toll-like receptor (TLR)-2 is considered the major receptor for Gram-positive bacteria in innate immunity, it does not play a major role in host defense against pneumococcal pneumonia. We wanted to investigate if in absence of an intact spleen as a first line of defense, the role of TLR2 during pneumococcal pneumonia becomes more significant, thereby explaining its insignificant role during infections in immune competent hosts.

**Methods:**

We intranasally infected splenectomized wildtype (WT), TLR2 knock-out (KO) and TLR2/4 double KO mice with either serotype 2 or 3 *S. pneumoniae*.

**Results:**

There were no differences between asplenic WT and TLR2KO mice of bacterial loads in lung homogenates and blood, cytokine and chemokine levels in the lungs, and lung pathology scores. TLR2/4 double KO mice were not impaired in bacterial control as well, which indicates that besides the interaction between *S. pneumoniae* and TLR2, the interaction between pneumolysin and TLR4 does not stimulate antibacterial defense in the asplenic host either.

**Conclusions:**

These results argue against a significant role of TLR2 in host defense during *S. pneumoniae* pneumonia in the asplenic state. Therefore, other components can provide sufficient backup mechanisms for TLR2 deficiency in the defense against intrapulmonary infections with *S. pneumoniae* of the otherwise immune competent host.

## Background

*Streptococcus pneumoniae* is the most frequently isolated pathogen in community acquired pneumonia [1,2]. Virtually all clinical *S. pneumoniae* isolates contain an external capsule consisting of repeating oligosaccharides. Based on antigenic differences in capsular polysaccharides, over 90 different serotypes of *S. pneumoniae* have been described. The capsule is the bacterium’s most important virulence factor, enabling the pathogen to evade recognition and phagocytosis by the host immune system [3,4]. In the host response to infection, the innate immune system mediates the first line of defense against invading pathogens, prior to the induction of the adaptive immune response. The family of Toll-like receptors (TLRs) prominently features in the innate immune system [5,6]. At present, 12 murine TLRs and 10 human TLRs have been identified. Each TLR recognizes distinct components of pathogens, referred to as pathogen-associated molecular patterns (PAMPs), thus composing an efficient way of sensing microorganisms [7]. Interaction of such a PAMP with a TLR is followed by a complex cascade of intracellular signalling, which leads to a proinflammatory response [5].

TLR2 has been designated the major receptor for Gram-positive bacteria since it signals the presence of lipoteichoic acid (LTA), peptidoglycan and lipopeptides, which are all components of the Gram-positive cell wall [5,6]. In accordance, viable and heat killed *S. pneumoniae* are primarily recognized by TLR2 [8,9] and TLR2 knockout (KO) mice did not develop airway inflammation upon intrapulmonary delivery of pneumococcal LTA *in vivo*[10]. Nonetheless, our laboratory and others have demonstrated that TLR2 does not play a major role in host defense against pneumococcal pneumonia [8,11,12]. Indeed, although TLR2KO mice displayed modestly reduced lung inflammation upon intranasal infection with *S. pneumoniae*, bacterial loads and mortality did not differ between TLR2KO and wild-type (WT) mice after intranasal inoculation with a wide range of infectious doses [8,11,12]. Furthermore, TLR2 did not contribute to an effective antibacterial defense during post-influenza pneumococcal pneumonia [13], which normally is associated with a much stronger inflammatory response in the lungs than primary pneumonia [14]. Together, these results suggest that other – TLR2 independent - components of host defense are sufficient to maintain an adequate immune response during respiratory tract infection caused by *S. pneumoniae*.

The spleen has an important role in innate as well as adaptive immunity. Especially the splenic marginal zone (MZ) is of great importance in innate immunity, since it contains MZ-macrophages and specific IgM-memory B cells that are capable of mounting a direct immune response against encapsulated bacteria such as *S. pneumoniae*, independently of the adaptive immune system [15,16]. Asplenia in humans is associated with an increased risk for lethal infections especially with *S. pneumoniae*[17-19], and multiple experimental studies have demonstrated that asplenic animals display a markedly impaired host defense response after infection with pneumococci as well [15,20-22].

Earlier investigations examined the role of TLRs in the interaction between splenocytes and *S. pneumoniae.* Incubation of WT splenocytes with intact *S. pneumoniae* rapidly induced TLR-dependent production of proinflammatory cytokines in the spleen [23]: deficiency of Myeloid differentiation primary response gene-88 (MyD88) adaptor protein, which signals all TLRs except TLR3, resulted in a complete loss of splenic cytokine and chemokine mRNA induction upon exposure to heat-killed *S. pneumoniae in vitro*. Notably, although other single TLRKO mice did not show reduced cytokine production, TLR2KO mice did have a loss of TNF-α and IL-1β secretion by macrophages and dendritic cells of the spleen [23]. After intraperitoneal injection of heat-killed pneumococci *in vivo*, however, TLR2KO mice displayed unaltered proinflammatory cytokine gene expression in their spleens, whereas MyD88KO mice had virtually completely lost their ability to mount a splenic cytokine response [24]. In contrast to its apparent insignificant role in the innate immune response in the spleen, TLR2 was shown to be important for the induction of a type 1 humoral immune response, as reflected by strongly diminished IgG3, IgG2a and IgG2b production in TLR2KO mice after intraperitoneal *S. pneumoniae* administration [24]. These findings suggest that the TLR2 mediated immune response during pneumococcal infection might partially be dependent on the spleen as an effector organ.

We here argued that in absence of an intact spleen as a first line of defense, the role of TLR2 during pneumococcal pneumonia becomes more important, thereby explaining the insignificant role for this pattern recognition receptor during respiratory tract infection by *S. pneumoniae* in the otherwise immune competent host. Therefore, to further elicit the role of TLR2, in the present study we compared the host response in splenectomized TLR2KO and WT mice after infection with encapsulated (serotype 2 and 3) *S. pneumoniae* via the airways.

## Methods

### Animals

Specific pathogen-free, 8–10 week old, C57BL/6 WT mice were purchased from Charles River (Maastricht, The Netherlands). TLR2KO mice (kindly provided by Shizuo Akira, Exploratory Research for Advanced Technology, Japan Science and Technology Agency, Suita, Osaka, Japan) were generated as described previously [25] and backcrossed to C57BL/6 background 6 times. TLR2/4 double KO mice were generated by crossing TLR2 [25] and TLR4KO mice [26], both backcrossed 6 times to a C57BL/6 background. All mice were bred in the animal facility of the Academic Medical Center in Amsterdam. In all experiments, male, age matched mice were used. All experiments were approved by the Animal Care and Use Committee of the University of Amsterdam (Amsterdam, Netherlands).

### Splenectomy

Mice were given buprenorphine (Temgesic^®^, Schering-Plough, Amstelveen, Netherlands) 0.075 mg/kg subcutaneously 15 minutes preoperatively, and anesthetized via inhalation of a mixture of O_2_ (1–2 l/min) and isoflurane 2.0-2.5% (Abbott, Kent, UK). A 1 cm incision was made in the left flank and peritoneum and the spleen was mobilized. In sham operated (Sham) mice the spleen was replaced. Splenectomy was performed after separately ligating the efferent and afferent vessels with Sofsilk 4–0 (Tyco Healthcare Group, Connecticut). 1 ml sterile saline was administered for fluid resuscitation in the abdominal cavity, and peritoneum and skin were closed with Vicryl 4–0 (Ethicon, Johnson&Johnson, Belgium). After 8 hours 0.05 mg/kg buprenorphine was administered. Mice were given a period of 2 weeks to recover after surgery before infection with *S. pneumoniae*.

### Bacteria

The *S. pneumoniae* strains used in this study were WT isolates D39 (serotype 2) and ATCC 6303 (American Type Culture Collection, Rockville, MD; serotype 3).

### Experimental design

Both *S. pneumoniae* strains were grown for 3–6 hours to mid-logarithmic phase at 37°C using Todd-Hewitt broth (Difco, Detroit, MI), supplemented with yeast extract (0.5%). Bacteria were harvested by centrifugion at 4000 rpm, and washed twice in sterile isotonic saline. For induction of pneumonia, bacteria were administered intranasally (total volume 50 μl) under light anaesthesia by inhalation of isoflurane (Abbott, Kent, UK) as described previously [27,28]. Infectious doses were as described in the Results section and table/figure legends. For determining bacterial loads, mice were sacrificed under isoflurane anaesthesia (2%/2 L) and samples were collected and processed as described [27,28]. Briefly, lungs and liver were homogenized at 4°C in 5 volumes of sterile isotonic saline with a tissue homogenizer (Biospect Products, Bartlesville, OK). Homogenates and blood were serially diluted 10-fold in sterile isotonic saline, and 50 μl volumes were plated onto sheep-agar plates and incubated over night at 37°C when colony forming units (CFU) were counted. Lung and liver homogenates were prepared for cytokine measurements in lysis buffer containing 300 mM NaCl, 30 mM Tris, 2 mM MgCl_2_.6H_2_O, 2 mM CaCl_2_.2H_2_O and 1% Triton X-100 (pH 7.4) with 0.5 ml protease-inhibitor (Roche Complete, 1 tablet protease inhibitor in 5 ml demi-water), incubated for 20 min. at 4°C, centrifuged at 3600 rpm for 10 min. and supernatants were stored at −20°C until assays were performed.

### Assays

Lung cytokines and chemokines (TNF-α, keratinocyte chemoattractant (KC/CXCL1), interleukin (IL)-1β and macrophage inflammatory protein 2 (MIP-2/CXCL2) were measured using specific ELISAs (R&D Systems, Minneapolis, MN) according to the manufacturers’ instructions.

### Histology

Lungs for histology were fixed in 4% formalin and embedded in paraffin. Five μm sections were stained with hematoxylin and eosin (HE). All slides were analyzed by a pathologist blinded for groups. To score lung inflammation and damage, the entire lung surface was analyzed with respect to the following parameters: bronchitis, edema, interstitial inflammation, intra-alveolar inflammation, pleuritis and endothelialitis. Each parameter was graded on a scale of 0 to 4, with 0 being ‘absent’ and 4 being ‘severe’. Total ‘lung inflammation score’ (TLIS) was expressed as the sum of the scores for each parameter, the maximum being 24. Granulocyte staining was done using FITC-labeled rat anti-mouse Ly-6 mAb (Pharmingen, San Diego, CA) as described earlier [8]. The entire lung surface was analyzed for Ly-6 G intensity by Image J (U.S. National Institutes of Health, Bethesda, MD, http://rsb.info.nih.gov/ij).

### Statistical analysis

Statistics were performed with GraphPad Prism version 4.00 for Windows, GraphPad Software, San Diego CA. Data are given as scatterplots or as means ± SEM.

Differences between groups were analyzed using Mann–Whitney U test. For survival analyses, Kaplan-Meier analysis, followed by a log rank test, was performed at different time points for the proportion of survivors. A value of p < 0.05 was considered statistically significant.

## Results

### TLR2 does not contribute to host defense during pneumonia caused by serotype 2 S. pneumoniae in splenectomized mice

**Figure 1:**
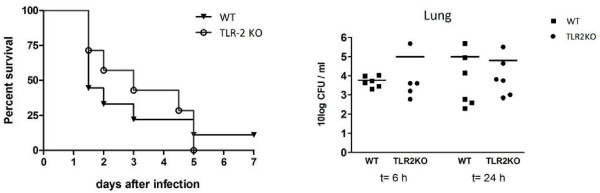
**TLR2 does not contribute to host defense against serotype 2** ***S. pneumoniae *after splenectomy.** Survival (**1A**) and bacterial outgrowth (**1B**) of WT mice and TLR2KO mice. Mortality was assessed two times per day after infection with 5*10^5^ CFU of *S. pneumoniae* (D39), for 1 week (8 WT versus 7 KO mice). Bacterial loads were determined 6 and 24 hours after infection with 6*10^5^ CFU. Data of bacterial loads are expressed as scatter-plots.

In otherwise immune competent mice, TLR2 deficiency does not influence mortality or bacterial growth during pneumococcal pneumonia [8,11,12]. We here investigated the impact of TLR2 on the outcome of pneumonia in splenectomized mice. For this purpose we infected WT and TLR2KO mice two weeks after splenectomy with *S. pneumoniae* D39 intranasally, at a dose known to be nonlethal to normal WT mice (5 x 10^5^ CFU) [12] and followed them for 1 week (Figure 1A). Although initially TLR2KO mice had a minor survival advantage, mortality did not significantly differ between WT and TLR2KO mice. We next determined bacterial loads in whole lung homogenates and blood at 6 and 24 hours after infection, i.e. at time points before the first mice started to die (Figure 1B). At both 6 and 24 hours, bacterial loads were identical in the lungs of WT and TLR2KO mice. In addition, the extent of dissemination of the infection did not differ between the two mouse strains: blood cultures were positive in 2 of 6 WT mice and 1 of 6 KO mice at 6 hours, at 24 hours after infection 4 of 7 WT mice and 5 of 6 TLR2KO mice had positive blood cultures (data not shown). These data demonstrate that even in the absence of a functional spleen TLR2 does not contribute to a protective immune response during pneumonia caused by a serotype 2 pneumococcus.

### TLR2 does not contribute to the inflammatory response during pneumonia caused by serotype 2 S. pneumoniae in splenectomized mice

**Figure 2:**
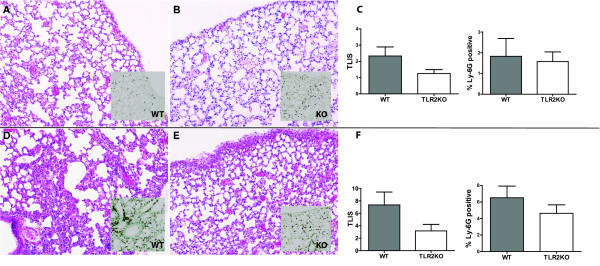
**Lung pathology induced by serotype 2** ***S. pneumoniae.*** Representative slides of lung tissue obtained at 6 hours (upper panels; **A**. WT and **B**. TLR2KO) and 24 hours (lower panels; **D**. WT and **E**. TLR2KO) after infection with 6*10^5^ CFU *S. pneumoniae* (D39)*.* Haematoxylin and eosin staining. Magnification 10x. *Insets*: representative slides of lung Ly-6 G staining (brown), showing influx of neutrophils. Findings are quantified by total pathology scores (total lung inflammation scores, TLIS) and scores of pulmonary Ly-6 G at 6 hours (**C**), and 24 hours (**F**) after induction of pneumococcal pneumonia. Data are expressed as means ± SE (3–7 mice per group).

Cytokines and chemokines are important in the antibacterial defense against *S. pneumoniae* pneumonia [2]. After splenectomy, an imbalance in cytokines has been proposed as possible mechanism for enhanced susceptibility to pneumococcal infections [29]. To investigate the role of TLR2 in the pulmonary inflammatory response in the asplenic host, we determined the concentrations of TNF-α, IL-1β, MIP-2 and KC in whole lung homogenates obtained at 6 and 24 hours after inoculation (Table 1). Except for MIP-2 at 6 hours after infection, there were no significant differences in the pulmonary levels of these mediators between TLR2KO and WT mice. To obtain further insight into a possible role of TLR2 in lung inflammation during pneumococcal pneumonia in splenectomized animals, we prepared lung tissue slides from TLR2KO and WT mice 6 and 24 hours after infection and determined semi-quantitative scores of specific histological alterations characteristic for bacterial pneumonia. These analyses showed a minor trend towards lower levels of lung pathology in TLR2KO mice at both time points, albeit non-significant. The extent of Ly-6 G positivity, indicating neutrophil influx, was similar in both mouse strains (Figure 2). Together, these data suggest that TLR2 does not contribute to the host response during serotype 2 *S. pneumoniae* pneumonia in splenectomized mice.

**Table 1:** **Lung cytokine and chemokine levels in splenectomized WT and TLR2KO mice, 6 and 24 h after infection with serotype 2** ***S. pneumoniae***

	**6 h**		**24 h**		
	**WT**	**TLR2KO**	**WT**	**TLR2KO**
TNF-α	1581 ± 268	1376 ± 190	1290 ± 49	1515 ± 249
IL-1β	173,5 ± 37	249,9 ± 91	132,9 ± 26	186,7 ± 35
MIP-2	2110 ± 333	4172 ± 919 *	951,6 ± 69	798,8 ± 53
KC	1244 ± 254	2680 ± 1825	1292 ± 241	1548 ± 263

Whole lung homogenates were obtained at 6 and 24 hours after intranasal infection with 6*10^5^ CFU of *S. pneumoniae* serotype 2 (D39). Data are means ± SEM (N = 6–7 per group).

* indicates p < 0.05 versus WT mice. TNF-α, IL-1β, MIP-2 and KC values are in pg/ml.

### TLR2 does not contribute to antibacterial defense during pneumonia caused by serotype 3 S. pneumoniae in splenectomized mice

**Figure 3:**
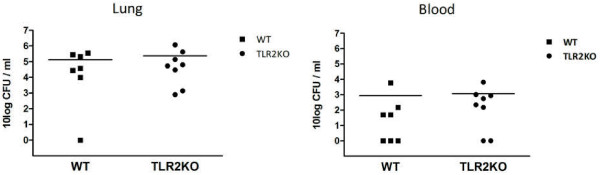
**TLR2 does not contribute to host defense against serotype 3** ***S. pneumoniae *after splenectomy.** Bacterial outgrowth after infection with serotype 3 *S. pneumoniae* (ATCC6303) in lungs and blood of WT mice and TLR2KO mice. Bacterial loads were determined 24 hours after infection with 7*10^4^ CFU. Data are expressed as scatter-plots.

To obtain further proof for an insignificant role of TLR2 during pneumococcal pneumonia in the asplenic host, we repeated part of the experiments described above with a serotype 3 *S. pneumoniae* (ATCC6303). For this purpose we intranasally infected TLR2KO and WT mice that had been splenectomized two weeks earlier with 7 x 10^4^ CFU *S. pneumoniae* ATCC6303, *i.e.* a dose expected to cause mortality in WT mice beyond the 24-hour time point [8,28] and determined bacterial loads in whole lung homogenates and blood 24 hours later. Similar to the experiments with the serotype 2 strain, there were no differences in bacterial loads in the lungs of WT and TLR2KO mice (Figure 3). In addition, bacterial loads in blood were not significantly different between groups; 4 of 7 WT mice had positive blood cultures compared to 6 of 8 TLR2KO mice (data not shown). Moreover, except for MIP-2, concentrations of TNF-α, IL-1β and KC in the lung homogenates obtained 24 hours after infection with serotype 3*S. pneumoniae* were not significantly different between WT and TLR2KO mice (Table 2).

**Table 2:** **Lung cytokine and chemokine levels in splenectomized WT and TLR2KO mice 24 hours after intranasal infection with serotype 3** ***S. pneumoniae***

	**WT**	**TLR2 KO**
TNF-α	1619 ± 277	1482 ± 536
IL-1β	261 ± 53	203 ± 75
MIP-2	2842 ± 216	2076 ± 216 *
KC	6767 ± 2446	4167 ± 1197

Whole lung homogenates were obtained at 24 hours after intranasal infection with 7*10^4^ CFU of *S. pneumoniae* serotype 3 (ATCC). Data are means ± SEM (N = 7–8 per group).

* indicates p < 0,05 versus WT mice, ** indicate p < 0,005 versus WT mice.

TNF-α, IL-1β, MIP-2 and KC values are in pg/ml.

**Figure 4:**
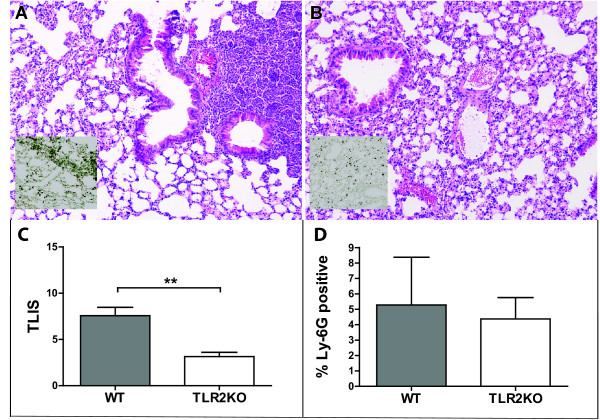
**Lung pathology induced by serotype 3** ***S. pneumoniae*.** Representative slides of lung tissue obtained at 24 hours after infection with 7*10^4^ CFU *S. pneumoniae* (ATCC), in WT mice (panel **A**) and TLR2KO mice (panel **B**).Representative slides of lung tissue obtained at 24 hours after infection with 7*10^4^ CFU *S. pneumoniae* (ATCC), in WT mice (panel A) and TLR2KO mice (panel B).Haematoxylin and eosin staining. Magnification 10x. *Insets*: representative slides of lung Ly-6 G staining (brown), showing influx of neutrophils. Findings are quantified by total pathology scores (total lung inflammation scores, TLIS) in panel **C**, and scores of pulmonary Ly-6 G in panel **D**, at 24 hours after induction of pneumococcal pneumonia. Data are expressed as means ± SE (3–7 mice per group). ** indicate p = 0,0012.

Lung pathology however, as reflected by total lung inflammation score, was significantly lower in TLR2KO mice as compared to WT mice (Figure 4); lungs of WT mice in general showed higher levels of interstitial inflammation, endothelialitis and pleuritis, whereas in KO mice there was less lung edema. This is in accordance with lower levels of lung pathology in TLR2KO mice infected with serotype 2 *S. pneumonia*. Neutrophil influx into the lungs, as reflected by percentages of Ly-6 G positive lung surface, did not significantly differ between strains.

### Splenectomized TLR2/TLR4 double KO mice display an unaltered host response during pneumococcal pneumonia

**Figure 5:**
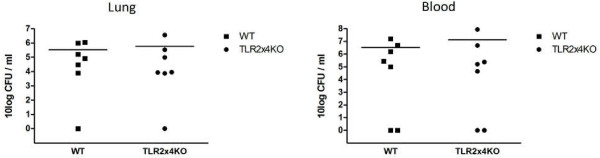
**TLR2 and TLR 4 do not contribute to host defense against *S. pneumoniae *after splenectomy.** Bacterial outgrowth after infection with serotype 2 *S. pneumoniae* (D39) in lungs and blood of WT mice and TLR2x4 double KO mice. Bacterial loads were determined 24 hours after infection with 4*10^5^ CFU. Data are expressed as scatter-plots.

Previous studies have suggested that TLR4 contributes to host defense against *S. pneumoniae* by virtue of its capacity to recognize pneumolysin [30,31]. Our laboratory recently demonstrated that TLR2 and TLR4 interact in the recognition of *S. pneumoniae* and that pneumolysin-induced TLR4 signalling can compensate for TLR2 deficiency during pneumococcal pneumonia [12]. We therefore considered it of interest to investigate whether TLR2/4 double KO mice have an altered immune response in the absence of a functional spleen. Thus we infected TLR2/4 double KO mice two weeks after splenectomy with 4 x 10^5^ CFU of serotype 2 *S. pneumoniae* (D39) and determined bacterial loads in whole lung homogenates and blood 24 hours later. Consistent with our findings in TLR2KO mice, there were no differences in bacterial loads in the lungs of TLR2/4 double KO and WT mice (Figure 5). Blood cultures were positive in 5 of 7 mice in both groups (data not shown). In addition, lung cytokine and chemokine concentrations obtained from lung homogenates 24 hours after infection with *S. pneumoniae* D39 did not differ between WT and TLR2/4 double KO mice (Table 3), and neither did lung pathology scores or neutrophil influx (Table 3). These results demonstrate that the combined action of TLR2 and TLR4 does not contribute to host defense during pneumococcal pneumonia in mice without a functional spleen.

**Table 3:** **Lung cytokine and chemokine levels and pathology scores in splenectomized WT and TLR2x4KO mice 24 hours after intranasal infection with serotype 2** ***S. pneumoniae***

	**WT**	**TLR2x4 KO**
TNF-α	1260 ± 209	1399 ± 227
IL-1β	226 ± 120	207 ± 62
MIP-2	1731 ± 290	2161 ± 542
KC	3597 ± 1536	6058 ± 3416
TLIS	7,2 ± 2,3	7,3 ± 0,8
Ly-6 G	3,3 ± 0,4	3,6 ± 1,0

Whole lung homogenates were obtained at 24 hours after intranasal infection with 4*10^5^ CFU of *S. pneumoniae*. Data are means ± SEM (N = 7 per group). TNF-α, IL-1β, MIP-2 and KC values are in pg/ml. TLIS = total lung inflammation score in arbitrary units, as described in the Methods section.

Ly-6 G score = percentage of lung surface that is Ly-6 G positive, see Methods section.

## Discussion

Previous studies have established that TLR2 does not contribute to an effective antibacterial defense during pneumococcal pneumonia [8,11-13], suggesting that other components of the immune system are sufficient to maintain an adequate response against *S. pneumoniae*. We here addressed the question whether an intact spleen, which plays an important role in the primary defense against pneumococci, can compensate for TLR2 deficiency during pneumococcal pneumonia, thereby explaining the insignificant role of TLR2 in the otherwise immune competent host. To this end, we compared the host response in asplenic WT and TLR2KO mice after infection with *S. pneumoniae* via the airways. We demonstrate that even in absence of the spleen, TLR2 does not contribute to host defense during pneumonia with serotype 2 or 3 *S. pneumoniae.*

Among the different TLR family members implicated in the immune recognition of *S. pneumoniae*, TLR2 sticks out as the most prominent [8,28,32]. In addition, killing and phagocytosis of *S. pneumoniae* by murine neutrophils has been reported to be impaired in the absence of TLR2 [33]. Nonetheless, the contribution of intact TLR2 signaling to protective immunity against the pneumococcus seems to depend on the localization of the primary infection: whereas TLR2 appears not essential for host defense during pneumonia [8,11-13], this receptor was reported to protect the host during meningitis caused by *S. pneumoniae*[34,35].

We here postulated that the potentially protective properties of TLR2 in host defense during pneumococcal pneumonia might become visible if another important line of defense (i.e. an intact spleen) would be eliminated. In line, our laboratory previously exposed a protective role for TLR2 during airway infection with a *S. pneumoniae* strain deficient for pneumolysin, an intracellular toxin recognized by TLR4, suggesting that during infection with WT *S. pneumoniae* TLR2 deficiency can be compensated for by pneumolysin-induced TLR4 signaling [12]. The present data clearly show that even in the hyper-vulnerable asplenic host TLR2 does not contribute to defense against pneumococcal pneumonia, as reflected by similar mortality and bacterial growth in TLR2KO and WT mice*.* Of note, even TLR2/4 double KO mice were not impaired in bacterial control, which indicates that besides the interaction between *S. pneumoniae* and TLR2, the interaction between pneumolysin and TLR4 does not stimulate antibacterial defense in the asplenic host either. We did not investigate non-TLR signaling in this model. Recently, it was shown that human and murine mononuclear cells respond to *S. pneumoniae* expressing pneumolysin by producing IL-1β via a mechanism that depended on the NOD-like receptor family, pyrin domain containing 3 (NLRP3) inflammasome. Specifically, release of IL-1β was induced by wild-type D39 *S. pneumoniae* but not by pneumolysin-deficient pneumococci [36], showing a TLR-4 independent route of pneumolysin signaling.

The current experiments were performed with two different *S. pneumoniae* serotypes (2 and 3). Although we did not find differences in bacterial loads between TLR2KO and WT mice after infection with either serotype, there was a consistent trend towards lower levels of inflammation in the lungs of TLR2KO mice, as determined by semi-quantitative pathology scores of lung tissue slides. Indeed, 6 and 24 hours after infection with serotype 2 *S. pneumoniae* pathology scores were lower in asplenic TLR2KO mice (albeit not statistically significant), whereas 24 hours after infection with serotype 3 *S. pneumoniae* TLR2KO mice displayed significantly less lung inflammation when compared to WT mice. These findings corroborate earlier studies from our laboratory demonstrating a role for TLR2 in the induction of lung inflammation early after induction of pneumococcal pneumonia in otherwise immune competent mice [8,12]. Lung cytokine and chemokine levels were not consistently influenced by TLR2 in asplenic mice, suggesting that other receptors, including other TLRs, are sufficient for induction of these inflammatory mediators.

We used an infectious dose that caused lethality in virtually all mice beyond the 48 hour time point. We specifically chose this dose considering that overwhelming pneumococcal infection after splenectomy in humans causes irreversible infection leading to mortality within the first 48 hours [17,18,37]. As a consequence, our data do not exclude a protective role for TLR2 in asplenic animals after infection with a low nonlethal dose of *S. pneumoniae*.

Previous studies have implicated TLR9 and MyD88 as important players in protective immunity in pneumococcal pneumonia [11,38]. We here focused on the role of TLR2 in defense during *S. pneumoniae* pneumonia in the asplenic host, considering that this TLR does not play a significant part in limiting bacterial growth in animals with an intact spleen [8,11,12]. Future studies are warranted to investigate the role of MyD88 and TLR9 in asplenic animals during respiratory tract infection caused by the pneumococcus.

## Conclusion

It has been well established that splenectomy renders the host very susceptible to infection with *S. pneumoniae*. The results presented here strongly argue against a significant role of TLR2 in host defense during *S. pneumoniae* pneumonia in the asplenic state. Therefore, in the immune competent host, there are other components of the immune system than the spleen that can provide a sufficient backup mechanism for TLR2 deficiency in the defense against intrapulmonary infections with *S. pneumoniae.*

## Competing interests

The authors declare that they have no competing interests.

## Author contributions

AJL performed the experiments, collected the data, performed analysis and drafted the article. AP helped with assays and prepared slides for pathology. OB and SF analyzed and scored all slides for histopathology. TP conceived the study design and revised the article.

## Pre-publication history

The pre-publication history for this paper can be accessed here:

http://www.biomedcentral.com/1471-2334/12/139/prepub
